# Ferroelectric polarization reversals in C_2_N/α-In_2_Se_3_ van der Waals heterostructures: a conversion from the traditional type-II to S-scheme

**DOI:** 10.3389/fchem.2023.1278370

**Published:** 2023-09-20

**Authors:** Yongle Zhong

**Affiliations:** Key Laboratory of Optoelectronic Devices and Systems of Ministry of Education and Guangdong Province, College of Physics and Optoelectronic Engineering, Shenzhen University, Shenzhen, China

**Keywords:** ferroelectric polarization reversal, semiconductor, heterostructure, S-scheme, first-principle calculations

## Abstract

**Introduction:** Ferroelectric substances, characterized by inherent spontaneous polarization, can boost photocatalytic efficiency by facilitating the separation of photogenerated carriers. However, conventional photocatalysts with perovskite-class ferroelectricity are generally constrained by their 3D arrangement, leading to less accessible active sites for catalysis and a smaller specific surface area compared to a 2D layout.

**Methods:** In my research, I developed a 2D ferroelectric heterostructure consisting of C_2_N/α-In_2_Se_3_. I performed first-principle calculations on the 2D C_2_N/α-In_2_Se_3_ heterostructure, specifically varying the out-of-plane ferroelectric polarization directions. I primarily focused on C_2_N/α-In_2_Se_3_ (I) and C_2_N/α-In_2_Se_3_ (II) heterostructures.

**Results:** My findings revealed that reversing the ferroelectric polarization of the 2D α-In_2_Se_3_ layer in the heterostructures led to a transition from the conventional type-II [C_2_N/α-In_2_Se_3_ (I)] to an S-scheme [C_2_N/α-In_2_Se_3_ (II)]. The S-scheme heterostructure [C2N/α-In_2_Se_3_ (II)] demonstrated a high optical absorption rate of 17% in visible light, marking it as a promising photocatalytic material.

**Discussion:** This research underscores the significance of ferroelectric polarization in facilitating charge transfer within heterogeneous structures. It provides a theoretical perspective for developing enhanced S-scheme photocatalysts, highlighting the potential of 2D ferroelectric heterostructures in photocatalytic applications.

## 1 Introduction

Photocatalysis is an emerging sustainable technology that utilizes semiconductor photocatalysts to drive chemical reactions by light irradiation. Upon photon absorption greater than the bandgap energy, photocatalysts generate electron-hole pairs that enable reduction-oxidation reactions on the surface. This allows the conversion of organic contaminants into less harmful products using solar energy (Wen et al., 2019). Titanium dioxide (TiO_2_) is the most studied photocatalyst owing to its chemical stability, low cost, and lack of toxicity (Maness et al., 1999). However, the large bandgap of TiO_2_ limits light harvesting to ultraviolet irradiation (Ma et al., 2016; Wang et al., 2018). Perovskite materials with the general formula of ABX_3_ have emerged as promising photocatalysts for various applications in recent years (Grabowska, 2016; Zhang et al., 2016). The prototypical perovskite photocatalyst is methylammonium lead triiodide (MAPbI_3_), which has a direct bandgap of 1.6 eV optimal for visible light absorption (He and Galli, 2014). The intrinsic properties of perovskites, including high optical absorption, a tunable bandgap, high charge carrier mobility, and long diffusion length, enable efficient generation and transportation of charge carriers upon light irradiation (Rehman et al., 2015). These merits make perovskites effective for photocatalytic redox reactions such as water splitting and CO_2_ reduction under solar light (Zhang et al., 2016; Huang et al., 2023). Strategies including elemental doping, heterostructuring with other semiconductors, and nanostructuring have been applied to further enhance the photocatalytic performance and stability of perovskites (Chen et al., 2020).

The recent surge of interest in fabricating van der Waals (vdW) heterostructures by layering various two-dimensional (2D) materials is a testament to the method’s effectiveness in engendering outstanding diverse properties (Frisenda et al., 2018; Huo and Guo, 2022; Ren et al., 2023a; Wang et al., 2023). These heterostructures benefit from in-plane stability, secured by robust covalent bonds, while layers in the stacking direction are interconnected through the weaker vdW forces (Ajayan et al., 2016; Zhang et al., 2023). This harmonious blend of the superlative properties of multiple 2D materials endows vdW heterostructures with superior electronic characteristics (Geim and Grigorieva, 2013). The large surface area provides abundant catalytic active sites (Rezaie et al., 2021). Tunable bandgaps by controlling thickness, strain, and stacking order allows optimized solar light absorption (Liu et al., 2016). High carrier mobilities facilitate charge transfer to reactive sites (Jiang et al., 2019; Ren et al., 2023b). Constructing heterostructures using 2D materials can promote charge separation and extend carrier lifetime. Hence, the exploration for stable vdW heterostructures with superior electronic properties has emerged as a hotbed of research (Huang et al., 2023).

Layered carbon nitride (C_2_N) has emerged as a promising metal-free photocatalyst for various energy conversion and environmental remediation applications (Huo and Guo, 2022). The graphitic structure of C_2_N with conjugated tri-s-triazine units linked by tertiary amines enables strong visible light absorption, high chemical stability, and tunable electronic properties. The creation of this 2D C_2_N material, facilitated by an efficient wet-chemical reaction method, opens new possibilities for its applications in photocatalysis (Zhang et al., 2015; Ashwin Kishore and Ravindran, 2017; Kishore and Ravindran, 2017). Recent studies have shown that modifying the textural and electronic properties of C_2_N can further enhance its photocatalytic (Kumar et al., 2018; Tang et al., 2019; Tan et al., 2021). Overall, rationally designed C_2_N-based photocatalysts have demonstrated great potential for solar fuel synthesis, CO_2_ reduction, nitrogen fixation, and water treatment (Yu et al., 2018; Zhou and Han, 2020). Further work to understand the structure-activity relationships and in-depth mechanisms in C_2_N photocatalysis could unlock its full potential for sustainable solar energy utilization.

The inherent polarization of ferroelectric materials unlocks new possibilities for engineering efficient photocatalysts by promoting the separation of photogenerated charge carriers. Xu et al. (2021) (Huang et al., 2018; Chen et al., 2019) demonstrated that the ferroelectric heterostructure of PbTiO_3_/g-C_3_N_4_ showed enhanced photocatalytic hydrogen production and RhB degradation, benefitting from deliberate manipulation of the ferroelectricity. As is well known, α-In_2_Se_3_ is a ferroelectric material. Computational and experimental analyses have demonstrated that emerging 2D α-In_2_Se_3_ can generate spontaneous ferroelectric polarization due to its unique asymmetric structure, which is considered a promising photocatalyst for water splitting and CO_2_ electrocatalytic reduction (Ju et al., 2019; Luo et al., 2020; Luo et al., 2021a; Ju et al., 2021). Recently, the COCN/α-In_2_Se_3_ heterostructure has achieved good results in the field of photocatalysis (Pan et al., 2023). In this paper, we employ first-principles calculations to present an in-depth exploration of a novel 2D vertical ferroelectric C_2_N/α-In_2_Se_3_ vdW heterostructure. C_2_N has a high recombination rate, which is not conducive to photolysis of water; researchers often use van der Waals methods to construct type-II to promote the separation of electrons and holes. The single-layer In_2_Se_3_ material has intrinsic out-of-plane ferroelectric properties. This kind of ferroelectric material can provide a built-in electric field, reduce the electron-hole recombination rate, and provide the possibility of S-scheme configuration. We can thus combine In_2_Se_3_ and C_2_N, and use ferroelectric polarization reversal to construct a paradigm van der Waals heterostructure to explore the potential of the heterostructure in photocatalytic activity (Luo et al., 2021b).

Our objective is to offer a comprehensive understanding of the electronic, optical, and polarization flip characteristics intrinsic to the heterostructure. By building upon the existing knowledge framework around 2D materials, we aspire to contribute significant insights to the design and optimization of vdW heterostructures with the ultimate goal of maximizing their potential in nanotechnology and optoelectronics applications.

## 2 Computational methods

All of the simulations are performed on the Quantum Espresso package (Giannozzi et al., 2009) based on density functional theory (DFT). The Perdew-Burke-Ernzerhof (PBE) (Perdew et al., 1996) exchange correlation functional at the generalized gradient approximation (GGA) level was used together with ultrasoft pseudopotentials (USPP) (Kresse and Joubert, 1999). The long-range vdW interlayer interaction in the C_2_N/α-In_2_Se_3_ heterostructure is described by Grimme’s DFT-D2 correction method (Grimme, 2006). The vacuum distance in *c*-direction was set 30 Å to avoid the interaction between the nearest neighboring units. The energy cutoffs of 60 and 600 Ry were chosen for the wave functions and the charge densities, respectively. During relaxation, the unit-cell lattice vectors, as well as the atomic coordinates, were fully relaxed until the force on each atom was less than 0.02 eV·Å^−1^ and the electron energy was less than 10^−6^ eV. The band-gap and the optical absorption are modified by the Heyd-Scuseria-Ernzerhof (HSE06) (Heyd et al., 2003) hybrid exchange-correlation functional. Other calculations are modified by the PBE exchange correlation functional at the generalized gradient approximation (GGA) level. Ground-state calculations were carried out on a regular 3 × 3 × 1 mesh of k points, and the Fermi surface was broadened by the Gaussian smearing method (Marzari et al., 1999).

## 3 Results and discussion

Before delving into the C_2_N/α-In_2_Se_3_ heterostructure, an investigation was conducted on the structural and electronic properties of individual C_2_N and α-In_2_Se_3_ monolayers. The C_2_N monolayer, belonging to the space group *P*6*mm* (183), features a uniform distribution of pores and nitrogen atoms. Distinguished by C-N separations of 1.33 Å and C-C separations of 1.42 and 1.46 Å, all the atoms within this single layer are positioned almost completely in the same plane. In the case of In_2_Se_3_, the most stable α- In_2_Se_3_ structure was analyzed, revealing a monolayer with the space group *Cm* (8). The optimized In-Se distance in the α-In_2_Se_3_ monolayer ranges from 2.54 to 2.91 Å. Optimized lattice parameters for both α-In_2_Se_3_ and C_2_N monolayers are 4.07 and 8.33 Å, respectively, which are constant with the experimental results of 4.05 and 8.30 Å (Mahmood et al., 2015; Zhou et al., 2015). These findings align with experimental measurements and previous theoretical outcomes.

**FIGURE 1:**
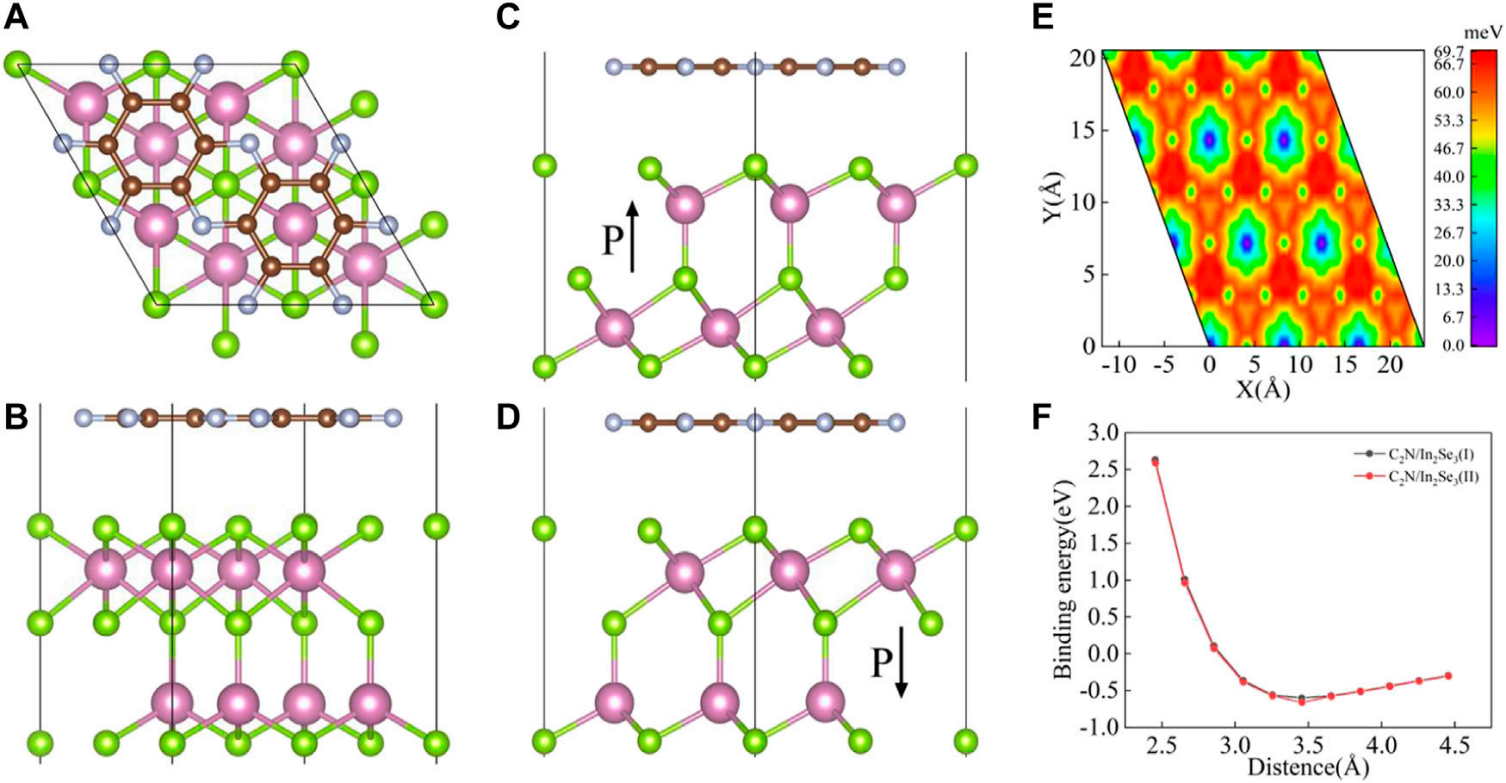
**(A)** The top view of the C_2_N/α-In_2_Se_3_ heterostructure structure. **(B)** The side view of the C_2_N/α-In_2_Se_3_ heterostructure structure. **(C)** The side view of the C_2_N/α-In_2_Se_3_ (I) heterostructure. **(D)** The side view of the C_2_N/α-In_2_Se_3_ (II) heterostructure. **(E)** The stacking-dependent potential energy surface of the C_2_N/α-In_2_Se_3_ heterostructure structure. **(F)** The binding energy evolution as a function of interlayer spacing.

Considering the lattice mismatch experimentally, a 1 × 1 C_2_N and 2 × 2 α-In_2_Se_3_ were adopted to construct a van der Waals heterostructure. The top and side views of optimized structure are illustrated in Figures 1A, B. Evident from Figure 1B, that α-In_2_Se_3_ monolayer displaies non-centro symmetryis and has a dipole moment in the *c*-direction (out-of-plane ferroelectric). Hence, there are two stacking models in which α-In_2_Se_3_ comes into contact with C_2_N to establish a heterostructure. Their structures are displayed in Figures 1C, D with opposite polarization directions. The structure in Figure 1C is defined as C_2_N/α-In_2_Se_3_ (I) and the structure in Figure 1D as C_2_N/α-In_2_Se_3_ (II). The lattice mismatch of the two C_2_N/α-In_2_Se_3_ heterostructures were calculated to be about 2.2%, enabling the construction of a matchable periodic interface for both structures. To get the lowest energy configuration, firstly, the minimum energy configuration in the *c*-direction was identified. Figure 1F shows the binding energy varies with layer spacing. The determination of the binding energy involves computations of the heterostructure’s energy and that of its monolayer, utilizing the equation $E_{b}=E_{C2N/\alpha -\text{In}2\text{Se}3}-E_{C2N}-E_{\alpha -\text{In}2\text{Se}3}$ to derive the differential. The binding energy trends of the two heterostructures closely mirror each other, initially presenting a parabolic decrease followed by a linear upswing. The binding energy of the two heterostructures exhibit nearly identical trends, first decreasing parabolically then increasing linearly. C_2_N/α-In_2_Se_3_ (I) possesses the lowest binding energy at a layer spacing of 3.455 Å. C_2_N/α-In_2_Se_3_ (II) possesses the lowest binding energy at 3.457 Å. Meanwhile, compared with C_2_N/α-In_2_Se_3_ (I), C_2_N/α-In_2_Se_3_ (II) had lower binding energy, which was the best configuration. The binding energies of the two are −604 and −660 meV respectively. The two E_b_ values are comparable to these of BlueP/g-GeC (−93.40 meV) (Gao et al., 2019) and much larger than that of BlueP/Sc_2_CF_2_ (−143.74 meV) (Li et al., 2020), indicating their good thermodynamic stability. We also investigated the influence of different stacking methods on the energy of C_2_N/α-In_2_Se_3_ heterostructure and calculated the stacking-dependent potential energy surface shown in Figure 1E, which illustrates how the energy varies with interlayer sliding. When the structure is most stable during interlayer sliding, each C_2_N atom aligns with an α-In_2_Se_3_ atomic gap. The lowest energy structure was found to be as shown in Figures 1A, B.

**FIGURE 2:**
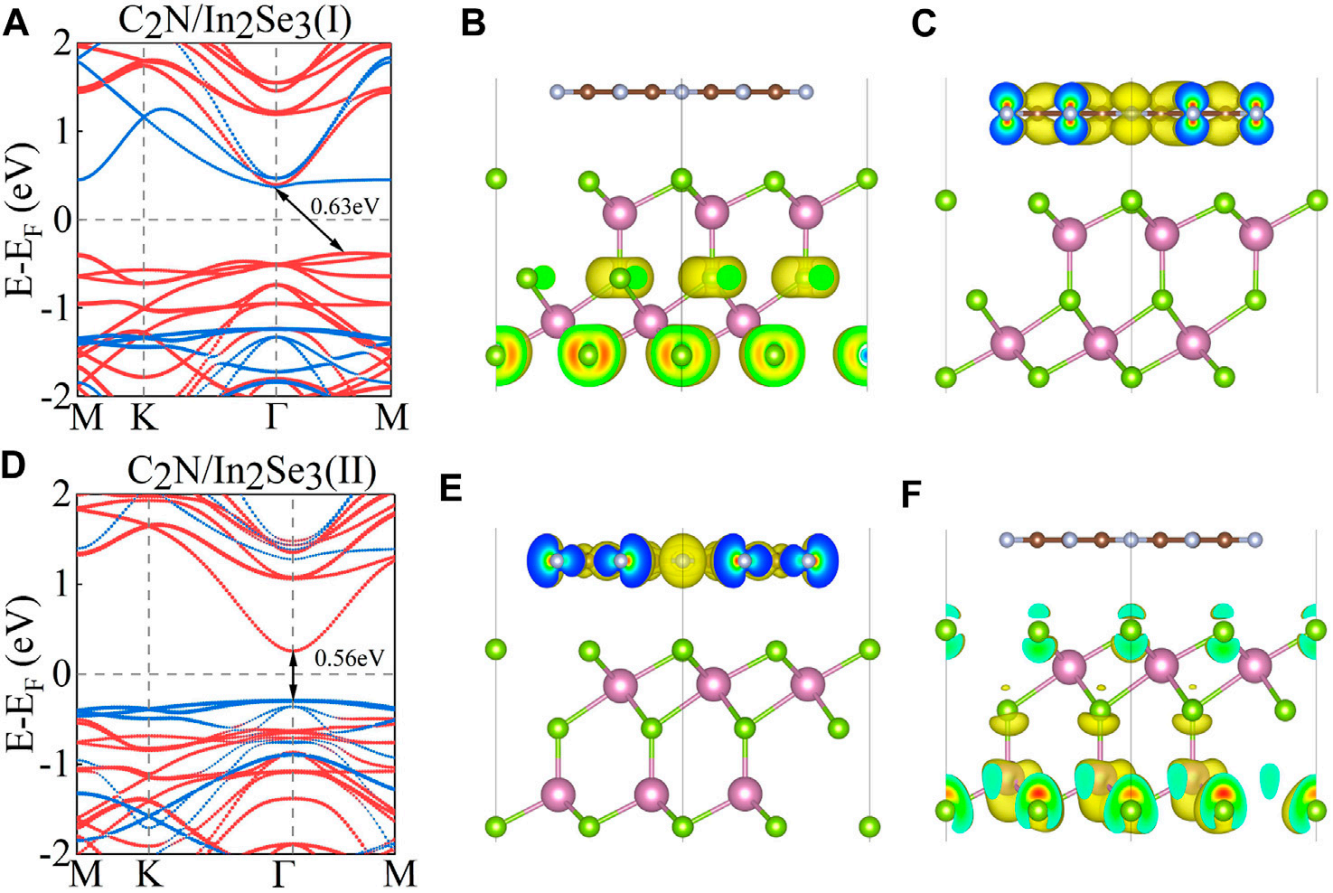
**(A,D)** The projected band structure of C_2_N/α-In_2_Se_3_ (I) and C_2_N/α-In_2_Se_3_ (II). The red line indicates the contribution of α-In_2_Se_3_, and the blue line represent the contribution of C_2_N. **(B,C)** The partial charge density of the valence band maximum (VBM) and conduction band minimum (CBM) for C_2_N/α-In_2_Se_3_ (I) with isosurface = 0.0014 e/Å^3^. **(E,F)** The partial charge density of the VBM and CBM for C_2_N/α-In_2_Se_3_ (II) with isosurface = 0.0012 e/Å^3^.

In the investigation of the electronic structure of C_2_N/α-In_2_Se_3_ heterostructures, the calculated projected band structures with PBE functional are presented in Figures 2A, D. A marked difference can be observed in the band structures of these two C_2_N/α-In_2_Se_3_ heterostructures. Detailed bandgap information and work function information are described in Table 1. In Figure 2A, the conduction band minimum (CBM) of C_2_N is lower than that of α-In_2_Se_3_, while the valence band maximum (VBM) of α-In_2_Se_3_ is higher than that of C_2_N. For the C_2_N/α-In_2_Se_3_ (II) heterostructure, however, both the CBM and VBM of C_2_N are higher than that of α-In_2_Se_3_, which is different to that of C_2_N/α-In_2_Se_3_ (I). But the two heterostructures maintain type-II band alignment. Furthermore, Figures 2B, C show the partial charge density of VBM and CBM for C_2_N/α-In_2_Se_3_ (I). Like the band structure of C_2_N/α-In_2_Se_3_ (I), the charge of CBM is localized on C_2_N, while the charge of VBM is localized on α-In_2_Se_3_. Upon reversal of the ferroelectric polarization in the α-In_2_Se_3_ layer [C_2_N/α-In_2_Se_3_ (II)], the contribution of CBM and VBM also switches. The partial charge density of C_2_N/α-In_2_Se_3_ (II) can be seen in Figures 2E, F, where the CBM and VBM are contributed to by C_2_N and In_2_Se_3_, respectively, which is opposite to that of C_2_N/α-In_2_Se_3_ (I). Interestingly, although the type-II band alignment is preserved, the contribution is now opposite to that of C_2_N/α-In_2_Se_3_ (I). Electrons and holes are separated in type-II band alignment and located in different layers of heterostructure, which greatly benefits photocatalytic devices (Ma et al., 2021; Ju et al., 2022).

**TABLE 1:** Lattice constants, bandgaps, and work functions for each structure.

	$a=b\left(Å\right)$	$E_{g}^{\text{PBE}}\left(\text{eV}\right)$	$E_{g}^{\text{HSE}}\left(\text{eV}\right)$	$W_{F1}^{\text{PBE}}\left(\text{eV}\right)$	$W_{F2}^{\text{PBE}}\left(\text{eV}\right)$
C_2_N	8.33	1.66	2.40	5.61	5.61
α-In_2_Se_3_	4.07	0.80	1.39	5.94	4.73
C_2_N/α-In_2_Se_3_ (I)	8.27	0.63	0.87	6.09	4.75
C_2_N/α-In_2_Se_3_ (II)	8.27	0.56	0.93	4.75	5.77

**FIGURE 3:**
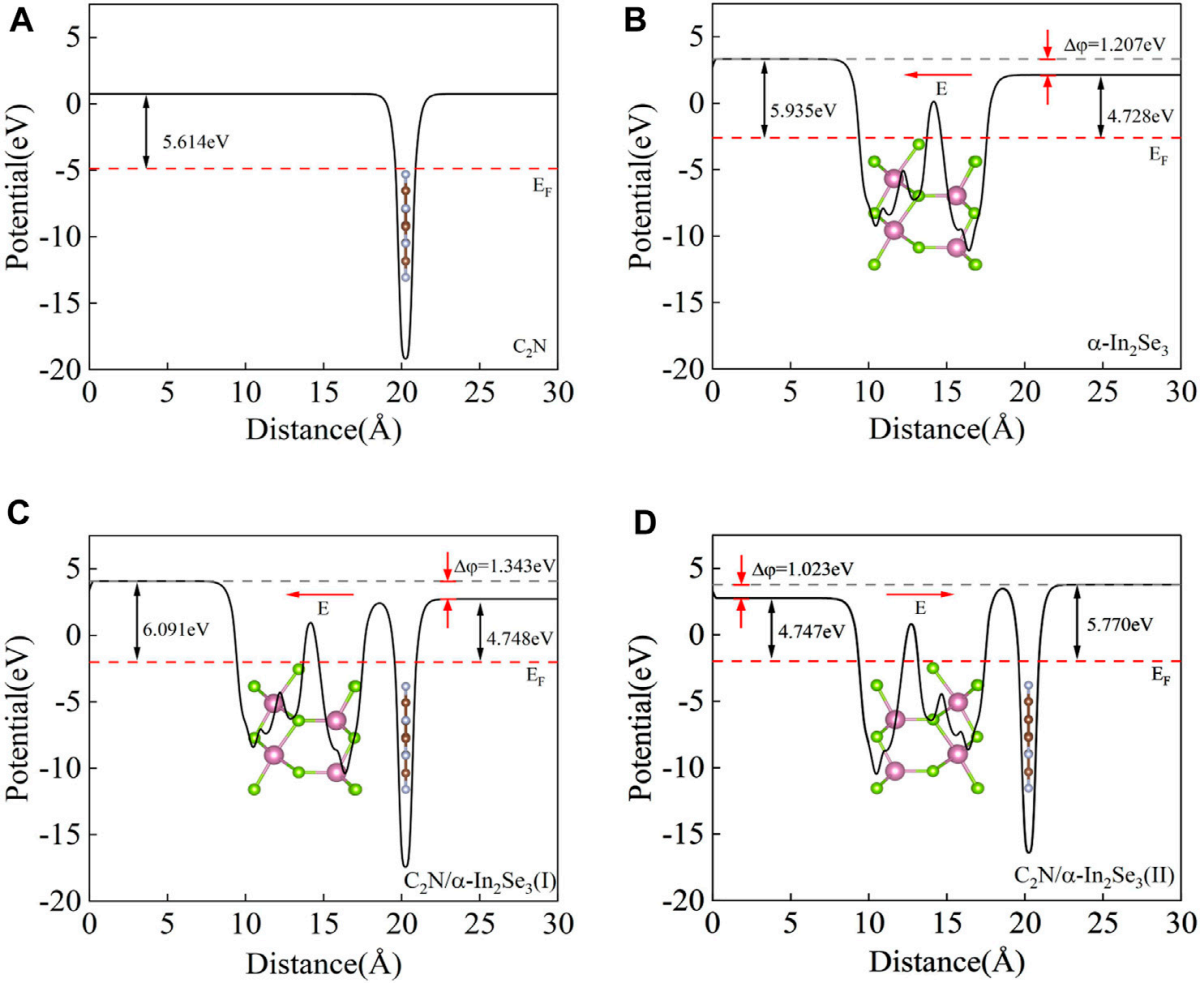
**(A)** The electrostatic potential of the C_2_N heterostructure. **(B)** The electrostatic potential of the α-In_2_Se_3_ heterostructure. **(C)** The electrostatic potential of the C_2_N/α-In_2_Se_3_ (I) heterostructure. **(D)** The electrostatic potential of the C_2_N/α-In_2_Se_3_ (II) heterostructure.

To obtain a thorough comprehension of the interfacial impact, an analysis was conducted on the average planar electrostatic potential along *c*-direction. The calculation of the work function is done by the formula $W_{F}=E_{v}-E_{F}$ . As depicted in Figure 3, the work function (W_F_) of the C_2_N monolayer is 5.61 eV, while the two surfaces of α-In_2_Se_3_ exhibit W_F_ of 4.73 and 5.94 eV. At the interface of the heterostructure, electrons typically migrate from the material with a lower W_F_ to the one with a higher W_F_. But when a two-dimensional material has different surface work functions on each side, charge transfer occurs when it contacts a two-dimensional material with the same surface work function on both sides. However, it should be noted that the charge transfer in this case may differ from that which would occur when the two materials are considered separately. Therefore, a comprehensive analysis of both the impact of the surface work function on each side of the two-dimensional material and the effects of charge transfer during material contact is necessary to gain a comprehensive understanding of the material’s electrical properties.

**FIGURE 4:**
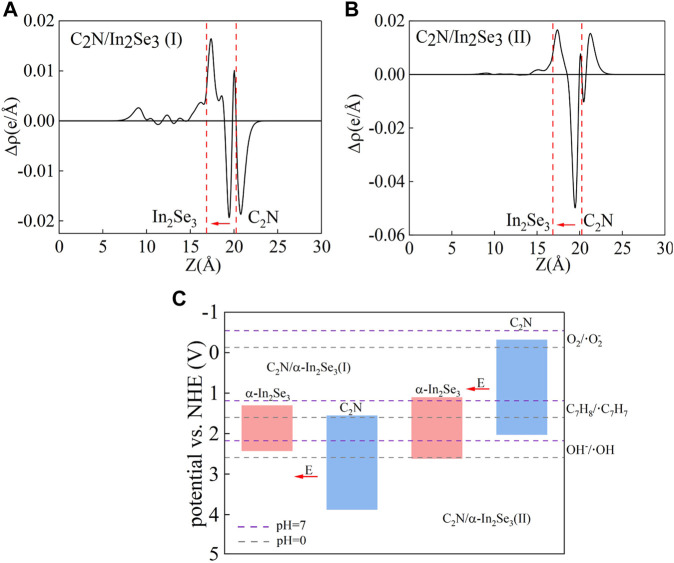
**(A,B)** The one-dimensional linear average differential charge density along the *c*-direction of C_2_N/α-In_2_Se_3_ (I) and C_2_N/α-In_2_Se_3_ (II). **(C)** The band edge alignments of the C_2_N/α-In_2_Se_3_ (I) and C_2_N/α-In_2_Se_3_ (II), and the solid red arrow shows the orientation of the inherent electric field.

To elucidate the interlayer charge transfer mechanism, differential charge density calculations were performed, accompanied by a one-dimensional linear average along the c-direction, as depicted in Figures 4A, B. Positive values indicate charge accumulation, while negative values signify charge decay. The direction of the electric field is from positive charges to negative charges. It can be found that no matter whether it is before or after ferroelectric inversion, charge accumulation occurs on the α-In_2_Se_3_ side and charge loss occurs on the C_2_N side. Notably, the direction of the built-in electric field remains unaltered before and after ferroelectric inversion, consistently pointing from C_2_N to α-In_2_Se_3_. Moreover, a difference W_F_ (Δφ) of 1.343 and 1.023 eV was observed in C_2_N/α- In_2_Se_3_ (I) and (II), respectively (Figures 3C, D), suggesting that the depolarization field in α-In_2_Se_3_ is either attenuated or amplified by varying interfacial interactions. When the built-in electric field aligns with the original electric field of α-In_2_Se_3_, Δφ is enhanced; otherwise, it weakens. Hindered by E_int_, the diffusive motion of electrons and holes eventually reaches equilibrium due to diffusion forces. This structure, as a result, effectively promotes carrier separation in C_2_N/α-In_2_Se_3_ heterostructures.

At the interface between the two semiconductors, the direction of the built-in electric field reflects the band bending at the interface. Based on Figure 4C, it can be inferred that upon photoexcitation, α-In_2_Se_3_ in C_2_N/α-In_2_Se_3_ (I) forms a potential well for photogenerated electrons and a potential barrier for photogenerated holes, while C_2_N in C_2_N/α-In_2_Se_3_ (I) forms a potential barrier for photogenerated electrons and a potential well for photogenerated holes. The drift and diffusion of photo-generated carriers in the valence band of α-In_2_Se_3_ and the conduction band of C_2_N are double constrained by the potential barrier and the built-in electric field and cannot be well recombined, but after the ferroelectric reversal occurs. In C_2_N/α-In_2_Se_3_ (II), though the potential barrier/well relationships remain unchanged, the built-in electric field facilitates the combination of photogenerated electrons in the α-In_2_Se_3_ potential well with photogenerated holes in the C_2_N potential well, leaving only photogenerated carriers obstructed by the potential barrier, leading to the formation of an S-scheme heterostructure. The C_2_N/α-In_2_Se_3_ (II) heterostructure meets simultaneous oxidation O_2_ (−0.13 V), C_7_H_8_ (1.60 V), and OH^−^ (2.59 V). In practice, the experiment is carried out in the non-zero pH solution according to the formula below:

$$E\left(\text{NHE}\right)=E\left(\text{RHE}\right)-0.0591\times \text{pH}$$ (1)

The catalytic potential at pH = 7 is also marked with a purple line in Figure 4C. It can be found that according to the specific reaction to be catalyzed, different pH environments can be selected to achieve the best results. S-scheme heterogeneous structures are more promising in photocatalysts compared to traditional type-II heterogeneous structures owing to their robust redox capabilities (Fu et al., 2019; Li et al., 2022; Li et al., 2023). Compared with the traditional type-II heterostructures, the photogenerated electrons and holes accumulate in the conduction band and valence band of the reduced semiconductor photocatalyst and the oxidized semiconductor photocatalyst, respectively, resulting in weakened redox ability. In the S-scheme heterostructure, the effective electrons and holes are preserved, and the meaningless photogenerated carriers are recombined.

**FIGURE 5:**
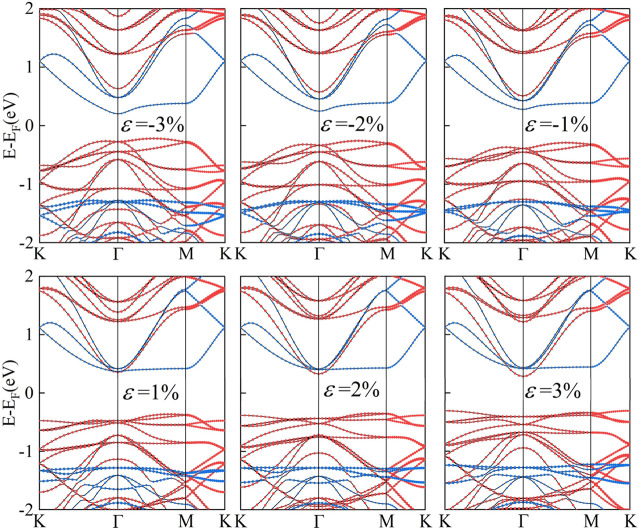
Demonstration of the band structure of C_2_N/α-In_2_Se_3_ (I) under different biaxial strains.

**FIGURE 6:**
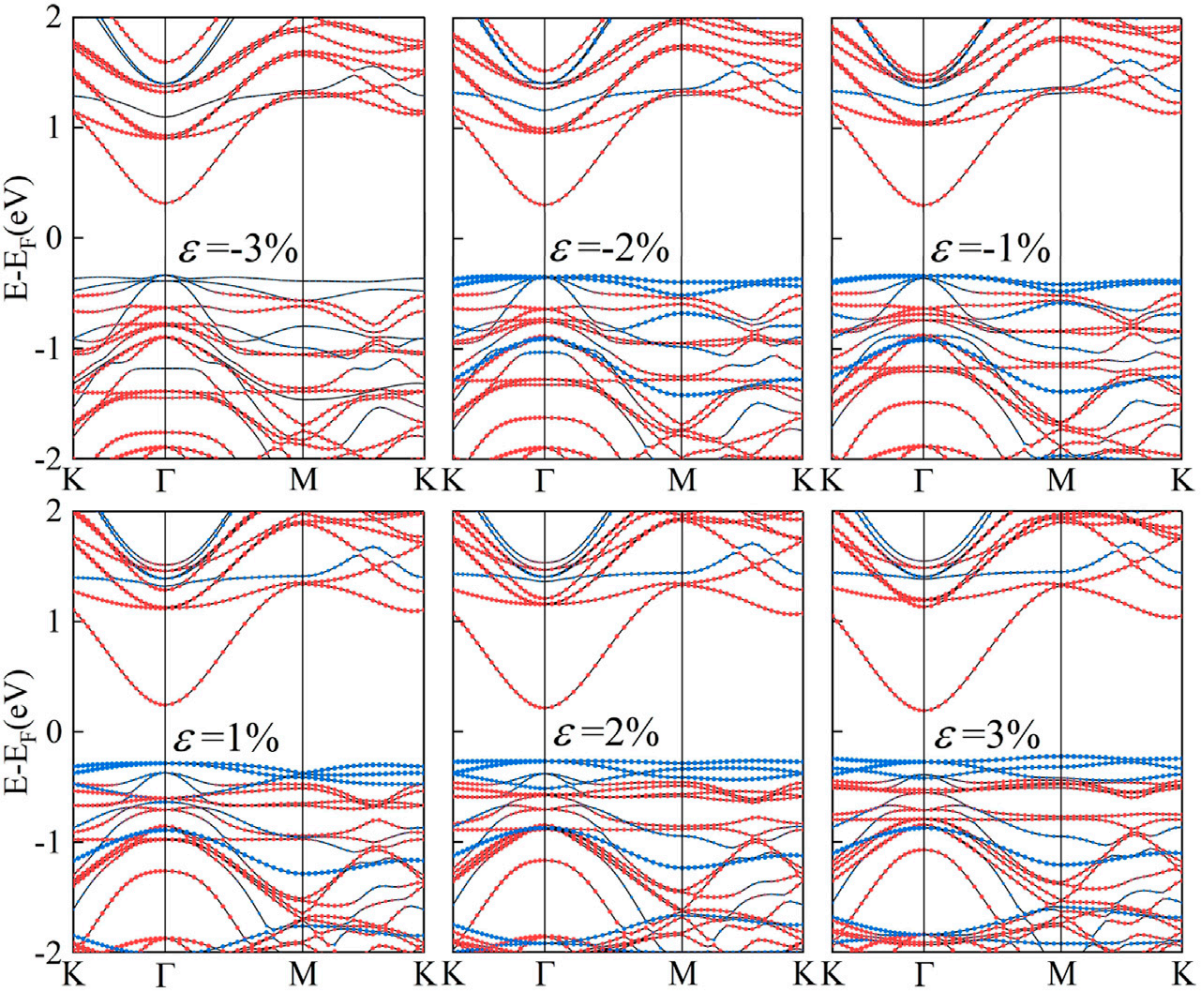
Demonstration of the band structure of C_2_N/α-In_2_Se_3_ (II) under different biaxial strains.

**FIGURE 7:**
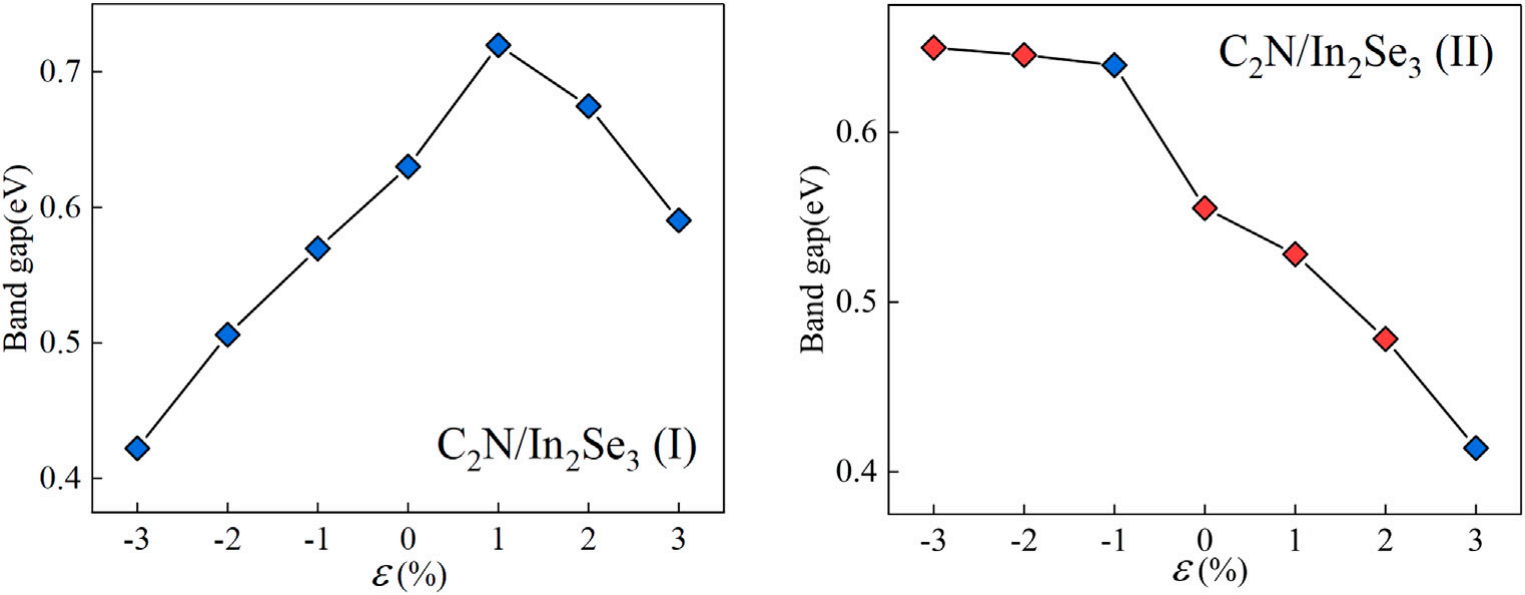
Demonstration of the band gap of C_2_N/α-In_2_Se_3_ (I) and under C_2_N/α-In_2_Se_3_ (II) different biaxial strains. Blue represents an indirect bandgap and red represents a direct bandgap.

Strain engineering, the deliberate introduction of strain in materials to modify their band structure, has emerged as an important tool in materials science (Hou et al., 2023). By manipulating the strain in a material, we can substantially alter its electronic, optical, and mechanical properties. Biaxial strain tuning was implemented on C_2_N/α-In_2_Se_3_ (I) and C_2_N/α-In_2_Se_3_ (II) heterostructures; the results are presented in Figures 5, 6, respectively. Under compressive strain, C_2_N/α-In_2_Se_3_ (I) retained a type-II band alignment. However, tensile strain transformed the heterostructure into a type-I alignment. In contrast, the C_2_N/α-In_2_Se_3_ (II) interface preserved a type-II band alignment under both compressive and tensile biaxial strains. Furthermore, the variation of bandgap size and bandgap type under the control of biaxial strain is demonstrated in Figure 7.

**FIGURE 8:**
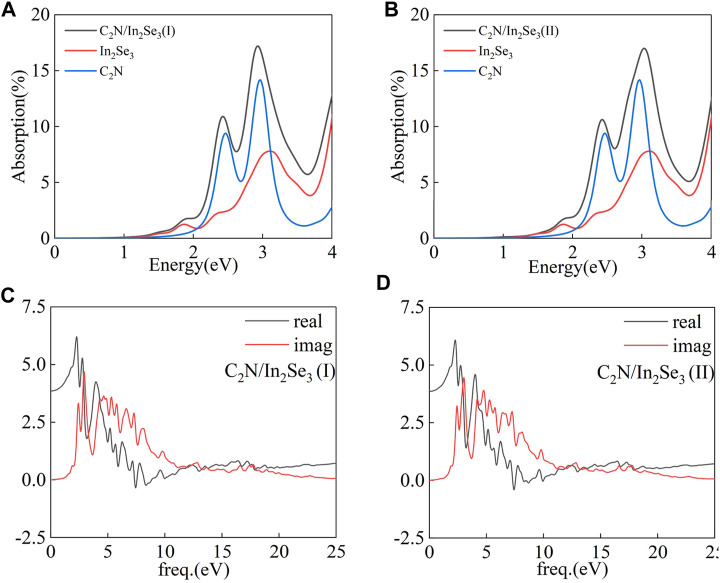
**(A,B)** The optical absorption of C_2_N/α-In_2_Se_3_ (I) and C_2_N/α-In_2_Se_3_ (II) heterostructures. **(C,D)** The dielectric function of C_2_N/α-In_2_Se_3_ (I) and C_2_N/α-In_2_Se_3_ (II) heterostructures.

The absorption coefficient is a critical factor in the S-scheme heterostructure. Therefore, the corresponding HSE absorption spectrums of C_2_N, α-In_2_Se_3_ monolayers, and C_2_N/α-In_2_Se_3_ heterostructures are shown in Figures 8A, B. The optical absorption coefficient α(ω) was obtained using the formula presented below:

$$\alpha \left(\omega \right)=\sqrt{2}\omega {\left[\sqrt{\varepsilon_{1}^{2}\left(\omega \right)+\varepsilon_{2}^{2}\left(\omega \right)}-\varepsilon_{1}\left(\omega \right)\right]}^{1/2}$$ (2)

where ε_1_(ω) and ε_2_(ω) are the real part and imaginary part of the complex dielectric function, respectively. The dielectric function varies with energy as depicted in Figures 8C, D. The real component characterizes the refractive properties of light, whereas the imaginary constituent signifies the absorbance qualities. Figure 8A displays the calculated optical absorption coefficient α(ω) as a function of energy for both the C_2_N/α-In_2_Se_3_ heterostructure and the respective isolated layers. As can be seen, the absorption coefficient of the C_2_N/α-In_2_Se_3_ (I) heterostructure initially rises with increasing phonon energy. The absorption coefficients of the C_2_N monolayer and C_2_N/α-In_2_Se_3_ (I) heterostructure exhibit prominent peaks in visible light. While the absorption coefficient of the α-In_2_Se_3_ monolayer gradually increases in visible light. It retains excellent optical absorption properties even after ferroelectric polarization reversal. The optical absorption property of C_2_N/α-In_2_Se_3_ (II) illustrates its potential in photocatalysts.

## 4 Conclusion

In summary, the optoelectronic characteristics of two-dimensional C2N/α-In2Se3 heterogeneous structures with varied polarization orientations in the α-In2Se3 layer were methodically examined using first-principles calculations. The results demonstrate that the traditional type-II [C_2_N/α-In_2_Se_3_ (I) heterostructure] with an indirect bandgap (0.63 eV) transformed into the promising S-scheme [C_2_N/α-In_2_Se_3_ (II) heterostructure] with a direct bandgap (0.56 eV) when the ferroelectric polarization of α-In_2_Se_3_ was reversed from up to down. The work function and one-dimensional linear average differential charge density revealed the S-scheme heterostructure is a promising photocatalytic material. Concurrently, excellent optical absorption was exhibited, providing design insights for novel photocatalysts.

## Data Availability

The raw data supporting the conclusion of this article will be made available by the authors, without undue reservation.
